# Restoring logic and data to phage-cures for infectious disease

**DOI:** 10.3934/microbiol.2017.4.706

**Published:** 2017-08-15

**Authors:** Philip Serwer

**Affiliations:** Department of Biochemistry and Structural Biology, The University of Texas Health Center, 7703 Floyd Curl Drive, San Antonio, Texas 28229-3900, USA

**Keywords:** phage characterization, phage propagation, phage stockpile, superbug

## Abstract

Antibiotic therapy for infectious disease is being compromised by emergence of multi-drug-resistant bacterial strains, often called superbugs. A response is to use a cocktail of several bacteria-infecting viruses (bacteriophages or phages) to supplement antibiotic therapy. Use of such cocktails is called phage therapy, which has the advantage of response to bacterial resistance that is rapid and not exhaustible. A procedure of well-established success is to make cocktails from stockpiles of stored environmental phages. New phages are added to stockpiles when phage therapy becomes thwarted. The scientific subtext includes optimizing the following aspects: (1) procedure for rapidly detecting, purifying, storing and characterizing phages for optimization of phage cocktails, (2) use of directed evolution in the presence of bacteriostatic compounds to obtain phages that can be most efficiently used for therapy in the presence of these compounds, (3) phage genome sequencing technology and informatics to improve the characterization of phages, and (4) database technology to make optimal use of all relevant information and to rapidly retrieve phages for cocktails that will vary with the infection(s) involved. The use of phage stockpiles has an established record, including a recent major human-therapy success by the US Navy. However, I conclude that most research is not along this track and, therefore, is not likely to lead to real world success. I find that a strong case exists for action to rectify this situation.

## 1. Basics

Antibiotic therapy for infectious disease is being compromised by emergence of multi-drug-resistant bacterial strains, often called superbugs. The alarm has been raised [1]–[5]. A response is to develop new antibiotics. But, in the long run, bacterial evolution of drug resistance is likely to outpace development of new antibiotics. Development of new antibiotics takes years; the rate of new antibiotic development is dropping [6],[7],[8]. Currently, ∼2,000,000 per year of superbug infections occur in the United States; ∼23,000 people die per year from these infections [9].

The logical response is to supplement antibiotic therapy with therapy via the following more rapidly changed agents: bacteria-infecting viruses (bacteriophages or phages). Phages can kill disease-causing bacteria while reproducing in them (phage therapy) [10]–[15]. Phage therapy pre-dates antibiotic therapy [10]–[15] and is more effective with some infections [11]. Phage therapy at the Eliava Institute (Tbilisi, Georgia, former USSR) often has a success rate above 90% [16]. The Eliava Institute and others [17] first collect a stockpile (sometimes called a library) of different phages. After diagnosing an infection, they draw optimal phages from the phage stockpile and administer a mixture (cocktail) of several of these phages. But, as with antibiotics, bacteria also become resistant to phages [10]–[13],[15].

When this occurs, the strategy is to make and use a cocktail of new phages. New phages are either newly isolated (in days) or drawn from the existing stockpile. If the stockpile is properly maintained, this strategy should be effective until a patient's immune system gains the upper hand. In the short term, the presence of more than one phage in a cocktail reduces problems with host resistance. Since bacteria co-exist with phages and new phages are isolated in days, logically, phage resistant bacteria will never, even in the long term, outpace phage therapy. Thus, phage therapy is a rational choice for superbug plagues, e.g., the mini-superbug plague of multi-drug-resistant *Clostridium difficile* [18]. Recent confirmation of this strategy is the dramatic success of US Navy investigators with a superbug *Acinetobacter* *baumannii* infection of a human [19]. The cure for this infection was use of two phage cocktails assembled from a stockpile [20] of *Acinetobacter* *baumannii* phages previously used for animals.

Phage therapy is also a logical response when antibiotic therapy is too drastic. For example, tooth decay (dental caries) is initiated primarily by acid produced by one or more of a few species of *Streptococcus* [21]. I think fair to state that preventing dental caries with daily, non-stop antibiotic therapy is a non-starter. On the other hand, using phage cocktail-rinses is a logical strategy, if the cocktail has the right phages. This example raises two related questions for all phage therapy. (1) What are the characteristics of the right phages? (2) How do we isolate these phages? Well accepted is that the right phages will be lytic, in contrast to lysogenic. Lytic phages are known to aggressively destroy host bacterial cells and not to insert their genomes into the host cell genome. Thus, lytic phages do not transfer toxin genes to bacteria; some lysogenic phages do this [10]–[15]. Lytic phages have genomes of double-stranded DNA. Some advances in improving phage isolation are mentioned below.

Otherwise, answers to the above questions are not known. Some mystery surrounds the most successful phage therapy experiences. Demystification is needed.

## 2. Scientific Subtext

For example, historically, studies of the phage therapy of human typhoid fever [22],[23] have produced anecdotal evidence that phages vary in their effectiveness. One statement (second paragraph of [22]) appears to be suggesting that small-plaque phages are relatively effective. My laboratory has shown [24] that small plaques in gels of the traditional concentration are one characteristic of relatively large (genome length > 200 kB) phages, sometimes called jumbo phages [25]. Indeed, for jumbo phages and others, plaque radius increases as the supporting gel concentration decreases [24],[26]; the slope of the plaque radius vs. gel concentration plot is a rough measure of the size of the phage [24]. But, apparently, this simple criterion (9 years old) has not been tested for selecting optimal phages for phage therapy cocktails.

Also, my laboratory has shown that some jumbo phages (1) do not propagate in the gels typically used for plaque formation, but do in others, and (2) do not continue propagation in liquid culture long enough to produce visible bursting (lysis) of the bacteria [24],[26],[27]. Apparently, plaque formation for these jumbo phages requires an adjuvant effect of the supporting gel. In general, phages of the following types are likely to be missed unless revised techniques are used: “(a) large and aggregating phages, (b) phages with long protruding fibers, (c) phages that adsorb to environmental particles and are released when in contact with a potential host bacterium, (d) phages that require hydrated polymer for aggressive propagation and (e) phages that typically exist in niches with other phages that outgrow them in conventional laboratory culture” [14].

However, as far as I know, phage therapy efforts have not taken account of these points. Thus, not surprising is that I think that current phage therapy efforts are far from optimized. Failure to find phages for some pathogens should be evaluated in this context.

This leads me to the final reason that phage therapy is needed. Surface infections typically include bacteria in semi-solid films called biofilms. These are typically resistant to antibiotics even if the bacteria, when outside of a biofilm, are not. Phage therapy is a possible response to biofilm-driven infections [14],[28].

## 3. Direction for the Future

Projected components of a future effort to further optimize phage therapy are the following.

(1) For each of the various pathogenic bacteria, build lytic phage stockpiles by using and improving updated procedures for both phage isolation/characterization and phage cocktail optimization. Among the most significant points are the use of (a) recent knowledge about jumbo phage isolation and propagation to optimize the collection of phages and adjuvants that can be used for cocktails (above), (b) directed evolution in the presence of bacteriostatic compounds (see [29]) to obtain phages that can be most efficiently used for therapy in the presence of these compounds, (c) advanced sequencing technology and informatics (examples [30],[31]) to improve the selection of phages used for a cocktail, and (d) advanced database technology to make optimal use of all relevant information and to rapidly retrieve phages for cocktails that will vary with the infection(s) involved.

In addition, we should determine whether this process will be made more efficient if we isolate/characterize phages on non-pathogenic bacteria before using them for pathogens. We should develop dry and room temperature storage of phages. This will simplify retrieval and delivery. We should optimize phage purification, which includes adjusting to the fact some (maybe most) jumbo phages are inactivated by centrifugation in cesium chloride density gradients (e.g., [32]). In summary, modern technology should be introduced to the making of phage cocktails.

At this point in history, efforts of the US Navy (and possibly affiliated groups) are the only Western (US and Western Europe) efforts that I see in the direction of past success and future optimization of phage therapy. This effort is aligned with the following aspect of the strategy of two leaders in the field of microbiology, Louis Pasteur [33],[34] and Félix d'Herelle [34]. The building of theory occurs via use of a direct approach to solving practical problems in microbiology. Both leaders bypassed the temptation to ask too many questions about how things work. Most additional questions are entertained during and after the solving of practical problems. This strategy avoids, for example, wasting time on determining which component of bad air causes malaria. Practical problem oriented strategy has similar potential in developing a phage-based, biological response to the systematic curing of metastatic cancer [14]. Thus, I find that mis-direction, well meaning though it may be, is a current problem in research on the practical use of phages.

The application to phage therapy is the following. The strategy discussed above, immediately under (1), is the only practical problem oriented strategy that currently exists. Any effort that draws resources away from implementation of this strategy is likely to be mis-direction and is certainly open to question. In any case, efforts of this type will not serve patients either now or in the near future.

Work of two types, frequent though it is, is also not likely to serve patients in the foreseeable future. The first type is recombinant phage construction. This endeavor is too slow and expensive for phage therapy, especially for responding to pathogens resistant to previous phage cocktails. More fundamentally, recombinant phage construction is typically based on one or a few variables. But, success of phage therapy depends on additional variables, some not even known and some likely de-optimized by the construction, especially when the phage enters a microbial community [35]. Phages isolated from the wild have undergone optimization for many variables (known and not known), potentially all relevant ones. No evidence exists that we now have (or will soon have) enough information for using human-design of phages to improve phage therapy.

By using Nature's phages, we will not only be clinically more successful, but we will be tapping into a genomic treasure when we determine the genomic sequences. Thus, (much) more science will get done than with recombinant phages. Maybe, we will eventually obtain enough information for future human-design of phages. Sociological backlash-derived issues would, however, have to be resolved first.

The second type is single phage isolation and *in vitro* testing without genomic sequencing and comparison to other phages for the same bacterial strain. This might be a good exercise for high school students. But, it contributes little if anything to phage therapy. Phage therapy depends on the isolation and rapid/inexpensive isolation/characterization/purification/storing/retrieval of hundreds of phages for each pathogen. A corollary is that we need a phage naming system that eliminates possible name duplication.

(2) Initiate clinical tests of phage therapy with human volunteers (These tests would be possible precursors to integrating phage therapy into general anti-microbial therapy). The volunteers would include people compromised by infections to the point to that either wellbeing or life is in danger. These infections include surface, biofilm-propagated infections, such as those encountered by diabetics, civilian victims of traumatic limb injury and military victims of traumatic limb injury. They also include drug resistant, typically hospital-acquired (often deadly) enteric infections. Change in current laws might be needed. Questions of finances and up scaling arise. Without discussing details, automation and personnel training are partial answers to these questions.

Finally, protocols are likely to be more complicated and variable than protocols for antibiotic therapy. Another anticipated update of phage therapy is use of a database, together with characteristics of the patient, to specify protocols. As more phages are isolated and information and its storage/retrieval advance, phage therapy may migrate to front-line status even for non-superbug infections. I find hard to imagine that any antibiotic acts more rapidly than some lytic phages, including coliphages T3 and T7. Phages T3 and T7 have 13–15 min life cycles (at 37 °C) and 50–100 progeny per host cell. In any case, clinicians must include people qualified to respond to possible negative or neutral effects of the therapy. Negative effects might include bacterial lysis that is rapid enough to generate toxicity [10],[11].

I typically do not like mixing politics with science. But, in the case of superbugs, this mixing appears necessary. Therapeutic success depends on the pursuit by investigators of logical directions in solving problems in the real world of microbiology and medicine, as did Louis Pasteur and, subsequently, Félix d'Herelle. To get this done, some political action may be necessary, especially by patients and their political representatives. Analogy exists with efforts that led to the various polio vaccines. I have previously reviewed USSR politics that assisted live polio vaccine use around the world [14]. A private foundation (March of Dimes), the result of a political effort spearheaded by an American president (Franklyn Roosevelt), was essential at all stages, especially at the beginning [36].

Antibiotic and other therapies eventually became commercialized, presumably because commercialization was the most efficient way to implement them. Commercialization was not successful with phage therapy in d'Herelle's time [34]. I think that renewed efforts at commercialization should be made, given subsequent and anticipated future advances in technology, as discussed above. Commercial advantages are suggested by the following. Cost recovery considerations cause drug companies to lose interest in developing limited life products, such as antibiotics. Longer times are expected to characterize amortization of costs of phage therapy infrastructure (not costs of one phage).
